# Secure Image Encryption Algorithm Based on Hyperchaos and Dynamic DNA Coding

**DOI:** 10.3390/e22070772

**Published:** 2020-07-15

**Authors:** Shuqin Zhu, Congxu Zhu

**Affiliations:** 1 School of Computer Science, Liaocheng University, Liaocheng 252059, China; shuqinzhu2008@163.com; 2 School of Computer Science and Engineering, Central South University, Changsha 410083, China

**Keywords:** five dimensional hyperchaos, image encryption, scrambling-diffusion, dynamic DNA coding, chosen-plaintext attack

## Abstract

In this paper, we construct a five dimensional continuous hyperchaotic system and propose an image encryption scheme based on the hyperchaotic system, which adopts DNA dynamic coding mechanism and classical scrambling diffusion encryption structure. In the diffusion stage, two rounds of diffusion are adopted and the rules of DNA encoding (DNA decoding) are dynamically changed according to the pixel value of the plaintext image, that is, the rules of DNA encoding (DNA decoding) used to encrypt different images are different, which makes the algorithm can resist chosen-plaintext attack. The encryption (decryption) key is only the initial value of the chaotic system, which overcomes the difficulty of key management in the “one time pad” encryption system. The experimental results and security analysis show that the algorithm has some advantages of large key space, no obvious statistical characteristics of ciphertext, sensitivity to plaintext and key and able to resist differential attacks and chosen plaintext attack. It has good application prospects.

## 1. Introduction

In recent years, with the rapid development of computer and network technologies, great changes have taken place in the means of communication; media communication has gradually become an important means of information exchange. However, it brings a huge hidden danger to multimedia communication for the openness and sharing of the network. Therefore, the security and confidentiality of images have become more and more important. The traditional encryption algorithms (such as DES, IDEA, etc.) are usually not suitable for image encryption for the high correlation and redundancy between adjacent pixels [1]. For the limitations of traditional algorithms, chaos shows its innate advantages as a new discipline. Chaos is a kind of deterministic but unpredictable nonlinear system, which has the characteristics of sensitivity to initial conditions and parameters, pseudorandomness and ergodicity. Therefore, chaos is closely related to cryptography and chaotic systems can be applied in cryptography, such as in the design of pseudo-random number generator (PRNG) [2] and S-Boxes [3]. Compared with traditional encryption algorithms, image encryption algorithm based on chaos theory has more advantages in security, complexity and speed [4,5,6,7,8,9]. In recent years, it has gradually become a hot research topic [10,11,12,13,14].

Generally, a chaotic cryptosystem consists of two stages—scrambling and diffusion. Scrambling means changing the position of image pixels, while diffusion means changing the pixel value and the combination of scrambling and diffusion can improve the security of encryption systems [15]. However, some cryptosystems are still cracked [16,17,18,19,20,21,22]. The reason is that the performance of chaos dynamics is not fully considered in the design of algorithms [19,20,21,23]. In order to overcome the shortcomings, hyperchaotic system can be applied to chaotic cryptography [23,24,25]. Generally speaking, hyperchaotic system has two or more positive Lyapunov exponents, more complex dynamic behavior and stronger randomness of the generated chaotic sequence [26]. On the one hand, hyperchaotic systems with two or more Lyapunov exponents are more sensitive to initial conditions, which can enhance the performance of a cryptosystem against differential attack. On the other, hyperchaotic systems have more variables and parameters, which can enlarge the key space of a cryptosystem. Therefore, image encryption with hyperchaos has higher security. But some encryption algorithms based on hyperchaotic systems are independent of the plaintext image and the output ciphertext image only depends on the key. Therefore, this kind of algorithm cannot resist the chosen-plaintext (ciphertext) attack [17,18,27].

In recent years, some cryptographic algorithms based on DNA coding have been proposed [28,29,30,31]. DNA molecule has the advantages of high information density, parallelism and ultra-low energy consumption and it has penetrated into the field of cryptography. The core of these algorithms is DNA coding and DNA computing, including DNA complementation, DNA addition, DNA subtraction and DNA XOR.

Gehani et al. proposed an image encryption method with “one-time pad” effect based on DNA coding, which can resist the attack of chosen-plaintext (ciphertext) [32]. Zhang et al. [31] proposed an image encryption algorithm based on DNA coding and two chaotic maps but Hermassi [33] pointed out that the algorithm proposed in Reference [31] has serious defects, that is, this encryption method is irreversible and cannot resist known plaintext attacks. Scanning the above image encryption algorithms based on DNA coding, they have the following security defects—first, the keys can be obtained through a pair of plaintext images and corresponding ciphertext images; second, the encryption process is not sensitive to the changes of plaintext images or keys. Third, the rules of DNA encoding and decoding are fixed. Therefore, in order to enhance security, this paper proposes an image encryption algorithm based on hyperchaos and dynamic DNA coding. The algorithm has three advantages. First of all, different DNA coding or decoding rules are adopted for pixels at different positions. Second, the generation of coding rules is related to the plaintext image, which makes the encryption algorithm have the encryption effect of “one time pad,” therefore, the algorithm can effectively resist the chosen-plaintext and known plaintext attacks. Third, although the algorithm has the encryption effect of “one time pad,” the key set of the algorithm is the initial value of the chaotic system and the encryption key used to encrypt different images is unchanged, while the hash value of an image is taken as a part of the keys in References [25,34,35] and the corresponding image hash value needs to be transferred when decrypting different images. In contrast, our algorithm does not need to transmit the secret key of hash value to the receiver, which leads to reduce the difficulty of key management.

The rest of this paper is organized as follows. Section 2 describes the construction and dynamics analysis of a new hyperchaotic system. Section 3 presents the DNA coding and key generating scheme. Section 4 proposes the image encryption scheme. Section 5 shows the experimental results of the proposed image encryption scheme and makes a security analysis. Section 6 completes the research paper with conclusions.

## 2. The Construction and Dynamics Analysis of a New Hyperchaotic System

In order to obtain a more complex hyperchaotic system for constructing the image encryption system, we will improve an existing 4D chaotic system and propose a new 5D hyperchaotic system.

### 2.1. The New 5D Hyperchaotic System

Considering the four-dimensional chaotic system proposed by Pan and Liu [36], as shown in expression (1)

(1) $$\{\begin{array}{l} \frac{dx}{dt}=a\left(y-x\right)\\ \frac{dy}{dt}=cy-xz+u\\ \frac{dz}{dt}=xy-bz\\ \frac{du}{dt}=-k_{1}x-k_{2}y \end{array}$$

Given (*a*, *b*, *c*, *k*_1_, *k*_2_) = (36, 3, 20, 2, 2), the four Lyapunov exponents of the chaotic system are λ_1_ = 1.4106, λ_2_ = 0.1232, λ_3_ = 0.0000, λ_4_ = −20.5339 and the dimension of Lyapunov is D_L_ = 3.0747.

Add a linear feedback control to the second equation of system (1) and we obtain a new five-dimensional hyperchaotic system, which is shown as

(2) $$\{\begin{array}{l} \frac{dx}{dt}=a(y-x)\\ \frac{dy}{dt}=c(x+y)-xz-pu\\ \frac{dz}{dt}=xy-bz\\ \frac{du}{dt}=my+fw\\ \frac{dw}{dt}=eyz \end{array}$$

**Figure 1:**
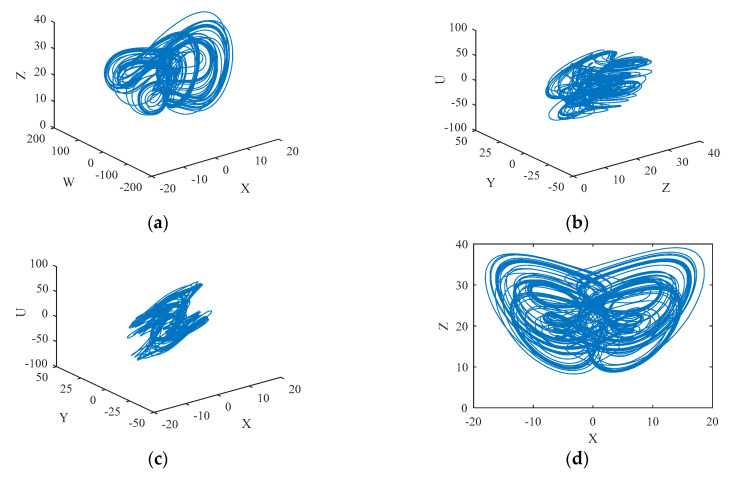

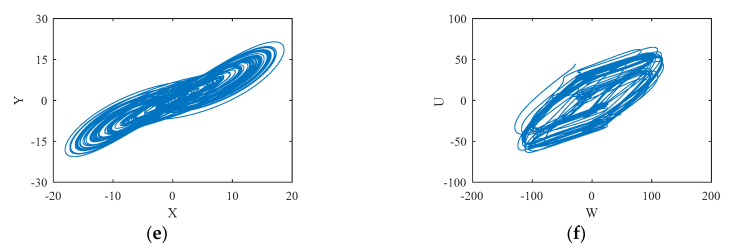
The trajectory of chaotic system (2). (**a**) X-W-Z space. (**b**) Z-Y-U space. (**c**) X-Y-U space. (**d**) X-Z plane; (**e**) X-Y plane. (**f**) W-U plane.

Given the parameters (*a*, *b*, *c*, *p*, *m*, *e*, *f*) = (25, 2, 10, 1, 16, 2, 1), the system (2) presents a chaotic state and the corresponding chaotic attractor is shown in Figure 1.

### 2.2. Dynamic Behavior Analysis of the New Hyperchaotic System

#### 2.2.1. Dissipativity

For system (2), take *a* = 25, *b* = 2, *c* = 10 and any *p*, *m*, *e*, *f*, we have

$$\Delta V=\frac{\partial \dot{x}}{\partial x}+\frac{\partial \dot{y}}{\partial y}+\frac{\partial \dot{z}}{\partial z}+\frac{\partial \dot{u}}{\partial u}+\frac{\partial \dot{w}}{\partial w}=-a-b+c=-17<0$$

In that case, the system is dissipative with an exponential contraction rate V_0_e^−(a+b−c)t^, which has nothing to do with *p*, *m*, *e*, *f*. This indicates that each volume containing the system orbit shrinks to zero as *t* → ∞ at an exponential rate −(*a* + *b* − *c*). Therefore, all system orbits are eventually limited to a subset of zero volumes.

#### 2.2.2. Equilibrium Point and Stability

Obviously, the origin *O*(0, 0, 0, 0, 0) is a unique equilibrium point. Linearizing system (2) at *O*(0, 0, 0, 0, 0) and the Jacobian matrix J is obtained as

$$J=\left[\begin{array}{ccccc} -a & a & 0 & 0 & 0\\ c & c & 0 & -p & 0\\ 0 & 0 & -b & 0 & 0\\ 0 & m & 0 & 0 & f\\ 0 & 0 & 0 & 0 & 0 \end{array}\right]$$

To gain its eigenvalues, let:

(3) |λI−J|=0

By solving Equation (3), the five eigenvalues of the matrix are obtained, which are *x*_1_ = −31.0190, *x*_2_ = 15.1689, *x*_3_ = 0.8501, *x*_4_ = −2, *x*_5_ = 0. The five characteristic roots are real roots but are not all negative. According to Routh-Hurwitz theorem, the equilibrium point is unstable and the possibility of the existence of chaotic attractor in the system is proved theoretically. The dynamic behavior of the system (2) affects the security of the proposed cryptosystem. In order to ensure the hyperchaotic property of system (2), we select the above parameters in the image encryption application.

#### 2.2.3. Lyapunov Exponents and Bifurcation

**Figure 2:**
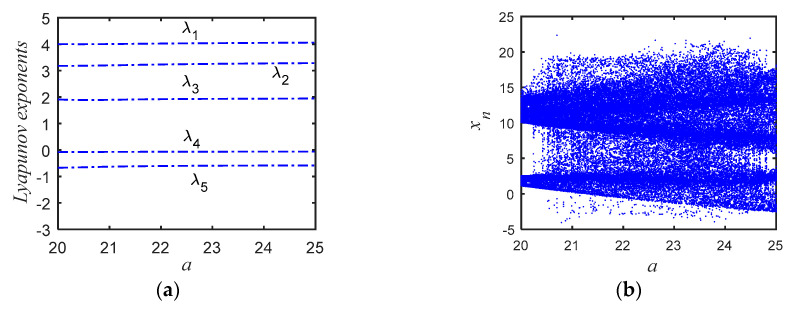
Lyapunov exponents and bifurcation of the system (2). (**a**) Lyapunov exponents versus parameter *a* for the initial values (2, 2, 2, 2, 2). (**b**) Bifurcation diagram of X versus parameter *a* for the initial values (2, 2, 2, 2, 2).

Figure 2a shows the calculation of Lyapunov exponents versus parameter *a* for the initial values (2, 2, 2, 2, 2) relative to the proposed system (2). When *a* = 25, it outputs the following values: λ_1_ = 4.0504, λ_2_ = 3.2641, λ_3_ = 1.8837, λ_4_ = −0.063394, λ_5_ = −0.56241. There are three positive Lyapunov exponents, means that the proposed system is a hyperchaotic attractor. Figure 2b shows the bifurcation diagram of *X* versus parameter *a* for the initial values (2, 2, 2, 2, 2).

## 3. DNA Coding and Key Generating Scheme

### 3.1. DNA Coding and Decoding Operation

A DNA sequence contains four nucleic acid bases A (adenine), C (cytosine), G (guanine) and T (thymine), where A and T are in a pair, while C and G are in another pair for the complementary property. In order to comply with the complement rule, there are eight kinds of coding schemes as listed in Table 1. In our DNA coding scheme, a pixel grayscale value of an image can be coded as a DNA sequence. The solution of our DNA coding scheme is implemented by the function DNAcode(pixel_value, code_rule), the parameter pixel_value represents the pixel value of an image, code_rule is an integer representing the coding rule. The return value of the function is a four-character string, which represents the corresponding DNA code. For example, the pixel value 180 has a binary representation ‘10110100’ and then the DNA sequence can be interpreted as ‘CTGA’ by encoding rule 2 in Table 1. DNAcode(180, 2) has the result of ‘CTGA’. Inversely, any DNA sequence with length 4 can be decoded as an 8-bit gray value. The solution of our DNA decoding scheme is implemented by the function DNAdecode(str_DNA, code_rule), the parameter str_DNA represents a four-character string pixel value of an image, code_rule is an integer representing the coding rule and the return value of the function is an 8-bit binary string. For instance, ‘GCCT’ is considered as ‘01101000’ by rule 8 and its decimal value is 104. DNAdecode(‘GCCT’, 8) has the result of ‘01101000′.

Table 2 shows the complementary property. The solution of the complementary property is implemented by the function DNAcomplement(str_DNA). DNAcomplement(‘A’) has the result of ‘T’, DNAComplement(‘T’) has the result of ‘A’, DNAComplement(‘G’) has the result of ‘C’, DNAComplement(‘C’) has the result of ‘G’.

### 3.2. The Key Generating Scheme

DNA coding rules are fixed in many image encryption algorithms based on chaos and DNA coding. However, there are only eight kinds of DNA coding rules. If the limited eight rules are used in a cryptographic system, they can be easily traversed one by one, which makes the algorithm weak in anti-exhaustive attack and easy to cause security risks. For this reason, this paper proposes a dynamic DNA coding method, that is, according to the random matrix generated by the chaotic system, the pixels at different positions are adopted different DNA coding rules in the eight DNA coding rules listed in Table 1. For example, B_1_ is a plain image matrix, R is a random matrix whose elements represent encoding rules and the first element of the matrix B_1_ is encoded with the first rule in Table 1 according to the matrix R. Namely, the pixel value 12 has a binary representation as ‘00001100’, then it can be encoded as ‘AATA’ by encoding rule 1 in Table 1. Therefore, the encoded matrix B_2_ is formed directly according to B_1_ and R.

$$B_{1}=\left[\begin{array}{ccc} 12 & 201 & 122\\ 122 & 133 & 98 \end{array}\right],\;R=\left[\begin{array}{ccc} 1 & 4 & 7\\ 2 & 3 & 5 \end{array}\right],\;B_{2}=\left[\begin{array}{ccc} AATA & CGTA & CAGG\\ GTCC & TCAA & TACA \end{array}\right].$$

#### 3.2.1. Generating Three Random Sequences CS, DS, K

Suppose the plaintext image PI is a gray-scale image with a size of *m* × *n*, then it is transformed into one-dimensional vector *Pt* = {*p_t_*(1), *p_t_*(2), …, *p_t_*(*L*)}, where *L* = *m* × *n*. The sequence *P_t_* is scrambled and output the permuted sequence *P* = {*p*(1), *p*(2), …, *p*(*L*)}. Then, *P* is encrypted with two rounds of diffusion process. In the first round of diffusion process, we encrypt *P* in the order of {*p*(*L*), *p*(*L*-1), …, *p*(1)} and output the temporary cipher sequence *CC* = {*cc*(1), *cc*(2), …, *cc*(*L*)}. In the second round of diffusion process, we encrypt *CC* in the order of {*cc*(1), *cc*(2), …, *cc*(*L*)} and output the final cipher sequence *C* = {*c*(1), *c*(2), …, *c*(*L*)}. Four random sequences CS, DS, K, R with length *L* are used in the two rounds of diffusion process. The following operational steps are the general methods for constructing pseudo-random key sequences in this paper. In order to facilitate the identification of variables and parameters in the following, we consider using {X_1_, X_2_, X_3_, X_4_, X_5_} instead the use of {X, Y, Z, U, W} for the system states, respectively.

**Step 1:** Generate five chaotic sequences. Given the initial values {*x*_1_(0), *x*_2_(0), *x*_3_(0), *x*_4_(0), *x*_5_(0)}, five chaotic sequences {X_1_, X_2_, X_3_, X_4_, X_5_} with length *L* = *m* × *n* are generated by solving the hyperchaotic system (1). We apply the fixed step technique to solve the system by using ode45( ) function in Matlab, the fixed step is 0.001. Where, X_1_ = {*x*_1_(*i*)|*i* = 1, 2, …, *L*}, X_2_ = {*x*_2_(*i*) |*i* = 1, 2, …, *L*}, X_3_ = {*x*_3_(*i*) |*i* = 1, 2, …, *L*}, X_4_ = {*x*_4_(*i*) |*i* = 1, 2, …, *L*}, X_5_ = {*x*_5_(*i*) |*i* = 1, 2, …, *L*}.

**Step 2:** Construction a sequence *S* = {*s*(1), *s*(2), …, *s*(*L*)} related to the current sequence of pixel values to be encrypted, which is used in the diffusion process. For the first round of diffusion process, *s*(*i*) are calculated by:

(4) $$s\left(i\right)=\{\begin{array}{l} 0,\;\mathrm{if}\;i=L\\ s(i+1)+p(i+1),\;\mathrm{if}\;i<L \end{array}$$

For the second round of diffusion process, *s*(*i*) are calculated by

(5) $$s\left(i\right)=\{\begin{array}{l} 0,\;\mathrm{if}\;i=1\\ s(i-1)+cc(i-1),\;\mathrm{if}\;i>1 \end{array}$$

**Step 3:** Generate two random coding sequence *CS* = {*c*s(1), *c*s(2), …, *c*s(*L*)} and random decoding sequence *DS* = {*d*s(1), ds(2), …, *d*s(*L*)} by combining *S* with chaotic sequence X_1_ and X_2_

(6) $$cs\left(i\right)=\mathrm{mod}(floor(\frac{s\left(i\right)\times x_{1}\left(i\right)}{256^{5}}\times 10^{12}),8)+1$$

(7) $$ds\left(i\right)=\mathrm{mod}(floor(\frac{s\left(i\right)\times x_{2}\left(i\right)}{256^{5}}\times 10^{12}),8)+1$$

where floor(X) rounds the elements of X to the nearest integers towards minus infinity. mod(X, Y) find the remainder of X divided by Y. *cs*(*i*) ∈ [1,8], *d*s(*i*) ∈ [1,8], *i* = 1, 2, …, *L. cs*(*i*) will be used as the DNA coding rule for *i*-th pixel and *d*s(*i*) will be used as the DNA decoding rule for *i*-th pixel, respectively. It can be seen from Equations (6) and (7) that the generation of random encoding (decoding) sequence is related to pixel values of the image to be encrypted.

**Step 4:** Generate a random sequence *K* = {*k*(1), *k*(2), …, *k*(l)} by combining the sequences *S* and X_3_

(8) $$k\left(i\right)=\mathrm{mod}(floor(\frac{s\left(i\right)\times x_{3}\left(i\right)}{256^{5}}\times 10^{12}),256)$$

From Equation (8), one can see that the sequence *K* = {*k*(1), *k*(2), …, *k*(*L*)} is related to the current sequence of pixel values to be encrypted. Sequence *K* will be used for XOR operation in our encryption scheme.

#### 3.2.2. Generating the Random Coding Rules R

The random coding rules R is a 0–1 sequence, R = {*r*(1), *r*(2), *r(*3), *r*(4)}, *r*(*j*) ∈ {0, 1}. We will use R for DNA complementary process on one pixel value. The generation steps are as follows.

**Step 1** According to the next formulas (9)–(12), the *i*-th random sequences *RR* = {*rr*(*i*, 1), *rr*(*i*, 2), *rr*(*i*, 3), *rr*(*i*, 4)} are generated by combining sequence *S* and chaotic sequence X_4_, X_5_.

(9) $$rr\left(i,1\right)=\mathrm{mod}(floor(\frac{s\left(i\right)\times x_{4}\left(i\right)}{256^{5}}\times 10^{12}),10)$$

(10) $$rr\left(i,2\right)=\mathrm{mod}(floor(\frac{s\left(i\right)\times x_{4}\left(i\right)}{256^{5}}\times 10^{13}),10)$$

(11) $$rr\left(i,3\right)=\mathrm{mod}(floor(\frac{s\left(i\right)\times x_{5}\left(i\right)}{256^{5}}\times 10^{12}),10)$$

(12) $$rr\left(i,4\right)=\mathrm{mod}(floor(\frac{s\left(i\right)\times x_{5}\left(i\right)}{256^{5}}\times 10^{13}),10)$$

**Step 2** Transform the sequence *RR* to a binary sequence *R* = {*r*(*i*, 1), *r*(*i*, 2), *r*(*i*, 3), *r*(*i*, 4)} as

(13) $$r\left(i,j\right)=\{\begin{array}{c} 0,\;if\;rr\left(i,j\right)\geq 5\\ 1,\;if\;rr(i,j)<5 \end{array},\;j=1,\;2,\;3,\;4.$$

## 4. The Image Cryptosystem

### 4.1. Encryption Algorithm

**Figure 3:**
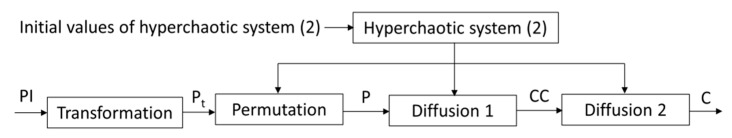
The overall flow chart of the cryptosystem.

The encryption algorithm includes one run of the permutation process and two rounds of the diffusion process. The overall outline of the proposed image encryption algorithm is shown in Figure 3.

**Figure 4:**
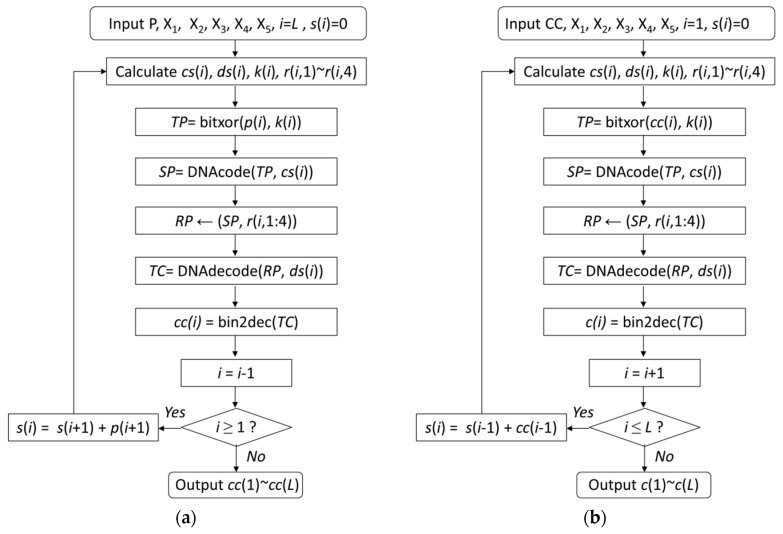
The flow chart of the first and second round of diffusion process. (**a**) The first round of diffusion process. (**b**) The second round of diffusion process.

The detail of the first and second round of diffusion process is shown in Figure 4.

#### 4.1.1. The Permutation Process

The steps of permutation process are as follows:

**Step 1**: Suppose the plain image PI is a gray-scale image with size of *m* × *n* and it is transformed into a 1D vector P*_t_* = {*p_t_*(1), *p_t_*(2), …, *p_t_*(*L*)}, where *L* = *m* × *n*.

**Step 2**: A new ordered sequence TX = {*tx*(*i*)|*i* = 1, 2, 3, …, *L*} is obtained by sorting the chaotic sequence {*x*1(1), *x*1(2), .., *x*1(*L*)} in ascending order and a random integer sequence SX = {*sx*(*i*)|*i* = 1, 2, 3, …, *L*} is generated by the position index of TX in X_1_. Where, 1 *≤ sx*(*i*) *≤ L*.

**Step 3**: According to the sequence SX, the sequence P*_t_* is scrambled and the permuted sequence is P = {*p*(1), *p*(2), …, *p*(*L*)} is obtained, where, *p*(*i*) are as

*p*(*i*) = *p_t_*(*sx*(*i*)), *i* = 1, 2, …, *L*. (14)

#### 4.1.2. The First Round of Diffusion Process

In the first round of diffusion process, the input pixel values P = {*p*(*i*)|*i* = 1, 2, …, *L*} are encrypted to output the temporary ciphertext sequence CC = {*cc*(*i*)|*i* = 1, 2, …, *L*}. The steps of the first round of diffusion process are as follows.

Input: P, X_1_, X_2_, X_3_, X_4_, X_5_.

Output: CC = {*cc*(*i*)|*i* = 1, 2, …, *L*}.

**Step 1**: Let *i* ← *L*, *s*(*i*) ← 0.

**Step 2**: Calculate *cs*(*i*), *ds*(*i*), *k*(*i*), *r*(*i*, 1), *r*(*i*, 2), *r*(*i*, 3), *r*(*i*, 4), by using Equations (6)–(13), respectively.

**Step 3**: Get the temporary ciphertext value *TP* corresponding to the pixel value *p*(*i*) as

(15) TP=bitxor(p(i),k(i))

where bitxor(x, y) indicates that x and y perform bitwise XOR operation. For example, TP = bitxor(255, 252) = bitxor(11111111_2_, 11111100_2_) = 0000 0011_2_ = 3.

**Step 4**: According to the random coding rule *cs*(*i*), the ciphertext value *TP* is encoded as a DNA code *SP* as

*SP* = DNAcode(*TP*, *cs*(*i*)) (16)

where DNAcode(▪, ▪) is the function described in the Section 3.1. *SP* is a four-character string and *SP* = {*sp*(1), *sp*(2), *sp*(3), *sp*(4)}.

**Step 5**: The DNA code *SP* is complemented to get the DNA code *RP* = {*rp*(1), *rp*(2), *rp*(3), *rp*(4)} according to the random sequence {*rr*(*i*, 1), *rr*(*i*, 2), *rr*(*i*, 3), *rr*(*i*, 4)} *as*

(17) $$rp\left(j\right)=\{\begin{array}{l} sp(j),\;\mathrm{if}\;r(i,j)=0\\ \mathrm{DNAcomplement}(sp(j)),\;\;\mathrm{if}\;r(i,j)=1 \end{array}$$

where *j* = 1, 2, 3, 4. DNAcomplement(▪) is the function described in the Section 3.1.

**Step 6**: According to the random decoding rule *ds*(*i*), the DNA code *RP* is decoded into a binary string *TC*

*TC* = DNAdecode(*RP*, *ds*(*i*)) (18)

where DNAdecode(▪, ▪) is the function described in the Section 3.1.

**Step 7**: Obtain the *i*-th cipher pixel value *cc*(*i*) by converting the 8-bit binary string *TC* into a decimal number, *cc*(*i*) = bin2dec(*TC*). Where, *y* = bin2dec(*x*) convert text representation of binary number *x* to decimal integer *y*.

**Step 8**: Let *i* ← *i*—1.

**Step 9**: If *i* ≥ 1, then *s*(*i*) ← *s*(*i* + 1) + *p*(*i* + 1) and repeat Step 2 to Step 8. If *i* < 1, then end the process.

After completing the first round of diffusion processing, we got the temporary ciphertext sequence *CC* = {c*c*(*i*)|*i* = 1, 2, …, *L*}.

#### 4.1.3. The Second Round of Diffusion Process

In the second round of the diffusion process, the input pixel values *CC* = {*cc*(*i*) | *i* = 1, 2, …, *L*} are encrypted to output the final cipher pixel values *C* = {*c*(*i*) | *i* = 1, 2, …, *L*}. The diffusion direction is from *i* = 1 to *i* = *L*. The steps of the second round of the diffusion process are as follows.

Input: CC, X_1_, X_2_, X_3_, X_4_, X_5_.

Output: C = {*c*(*i*) | *i* = 1, 2, …, *L*}.

**Step 1**: Let *i* ← 1, *s*(*i*) ← 0.

**Step 2**: Calculate *cs*(*i*), *ds*(*i*), *k*(*i*), *r*(*i*, 1), *r*(*i*, 2), *r*(*i*, 3), *r*(*i*, 4), by using Equations (6)–(13), respectively.

**Step 3**: Get the temporary ciphertext value *TP* corresponding to the pixel value *p*(*i*) with *k*(*i*) as TP=bitxor(cc(i),k(i)).

**Step 4** to **Step 6** are the same of the first round diffusion process.

**Step 7**: Obtain the *i*-th cipher pixel value *c*(*i*) by converting the 8-bit binary string *TC* into a decimal number, *c*(*i*) = bin2dec(*TC*).

**Step 8**: Let *i* ← *i* + 1.

**Step 9**: If *i* ≤ *L*, then *s*(*i*) ← *s*(*I* − 1) + *cc*(*I* − 1) and repeat Step 2 to Step 8. If *i* > *L*, then end the process.

After completing the second round of diffusion processing, we got the final ciphertext sequence *C* = {c(*i*) | *i* = 1, 2, …, *L*}. Transform *C* into a 2D matrix with size *m* × *n*, then the final encrypted image is obtained.

### 4.2. Decryption Algorithm

A feasible encryption algorithm should be reversible, that is, it should be decrypted by the one who has the right key. The approximate operation steps are as follows.

#### 4.2.1. The First Round of Inverse Diffusion

In the first round of inverse diffusion, we recover the temporary ciphertext sequence CC = {*cc*(*i*) | *i* = 1, 2, …, *L*} from the final ciphertext sequence C = {*c*(*i*) | *i* = 1, 2, …, *L*}. From Equation (5), we already know that *s*(1) = 0. Therefore, we can recover the first pixel value *cc*(1) at first. Then, we can derive *s*(2) from *s*(1) and *cc*(1) and use *s*(2) to recover the pixel value *cc*(2), … and so on, we can derive *s*(*L*) from *s*(*L*-1) and *cc*(*L*-1) and use *s*(*L*) to recover the pixel value *cc*(*L*). The concrete operational steps of the first run of inverse diffusion are as follows.

Input: C, X_1_, X_2_, X_3_, X_4_, X_5_.

Output: CC = {*cc*(*i*) | *i* = 1, 2, …, *L*}.

**Step 1**: Let *i* ← 1, *s*(1) ← 0.

**Step 2**: To calculate *cs*(*i*), *ds*(*i*), *k*(*i*), *r*(*i*, 1), *r*(*i*, 2), *r*(*i*, 3) and *r*(*i*, 4) by using Equations (6)–(13), respectively.

**Step 3**: Calculate DNA code *RP* by

*RP* = DNAcode(*C*(*i*), *ds*(*i*)) (19)

where DNAcode(▪, ▪) is the function described in the Section 3.1.

**Step 4**: The DNA code *RP* is complemented to get the DNA code SP = {*sp*(1), *sp*(2), *sp*(3), *sp*(4)} according to the random sequence {*r*(*i*, 1), *r*(*i*, 2), *r*(*i*, 3), *r*(*i*, 4)} as

(20) $$sp\left(j\right)=\{\begin{array}{l} rp(j),\;\mathrm{if}\;r(i,j)=0\\ \mathrm{DNAcomplement}(rp(j)),\;\;\mathrm{if}\;r(i,j)=1 \end{array}$$

where *j* = 1, 2, 3, 4. DNAcomplement(▪) is the function described in the Section 3.1.

**Step 5**: According to the random coding rule *cs*(*i*), the ciphertext value *TP* is encoded as a DNA code *SP* as

*TC* = DNAdecode(*SP*, *cs*(*i*)) (21)

where DNAdecode(▪, ▪) is the function described in the Section 3.1.

**Step 6**: Converting the 8-bit binary string *TC* into a decimal number *TP*, *TP* = bin2dec(*TC*).

**Step 7**: Recover the temporary ciphertext value *cc*(*i*) by

*cc*(*i*) = bitxor(*TP*, *k*(*i*)) (22)

**Step 8**: *i* ← *i* + 1.

**Step 9**: If *i* ≤ *L*, then

*s*(*i*) ← *s*(*I* − 1) + *cc*(*i* − 1) (23)

and repeat Step 2 to Step 8. If *i* > *L*, then end the process.

After completing the first round of inverse diffusion processing, the temporary ciphertext sequence CC = {c*c*(*i*) | *i* = 1, 2, …, *L*} is obtained.

#### 4.2.2. The Second Round of Inverse Diffusion

In the second round of inverse diffusion process, the input pixel values *CC* = {*cc*(*i*) | *i* = 1, 2, …, *L*} are decrypted to recover the plain pixel values *P* = {*p*(*i*) | *i* = 1, 2, …, *L*}. From Equation (4), we already know that *s*(*L*) = 0. Therefore, we can recover the first pixel value *p*(*L*) at first. Then, we can derive *s*(*L*-1) from *s*(*L*) and *p*(*L*) and use *s*(*L*-1) to recover the pixel value *p*(*L* − 1), … and so on, we can derive *s*(1) from *s*(2) and *p*(2) and use *s*(1) to recover the pixel value *p*(1). The concrete operational steps of the second round of inverse diffusion are as follows.

Input: CC, X_1_, X_2_, X_3_, X_4_, X_5_.

Output: P = {*p*(*i*) | *i* = 1, 2, …, *L*}.

**Step 1**: Let *i* ← *L*, *s*(*L*) ← 0.

**Step 2**: To calculate *cs*(*i*), *ds*(*i*), *k*(*i*), *r*(*i*, 1), *r*(*i*, 2), *r*(*i*, 3) and *r*(*i*, 4) by using Equations (6)–(13), respectively.

**Step 3**: Calculate DNA code *RP* by

*RP* = DNAcode(*cc*(*i*), *ds*(*i*)) (24)

where DNAcode(▪, ▪) is the function described in the Section 3.1.

**Step 4** to **6** are exactly the same as **Step 4** to **6** of the first round of inverse diffusion.

**Step 7**: Recover the plaintext value *p*(*i*) by

*p*(*i*) = bitxor(*TP*, *k*(*i*)) (25)

**Step 8**: Let *i* ← *i* − 1.

**Step 9**: If *i* ≥ 1, then

*s*(*i*) ← *s*(*i* + 1) + *p*(*i* + 1) (26)

and repeat Step 2 to Step 8. If *i* < 1, then end the process.

After completing the second round of the inverse diffusion processing, the pixel value sequence P = {*p*(*i*) | *i* = 1, 2, …, *L*} is obtained.

#### 4.2.3. Inverse Permutation Process

To recover the plain image pixel sequence P*_t_* = {*p_t_*(1), *p_t_*(2), …, *p_t_*(*L*)} from the sequence P = {*p*(1), *p*(2), …, *p*(*L*)}. The concrete operations are the inverse operations of Equation (14), which are as follows

*p_t_*(*sx*(*i*)) = *p*(*i*), *i* = 1, 2, …, *L*. (27)

Our encryption algorithm is also suitable for color image. Assume the size of the color plain image is *m* × *n* × 3, decompose it into its red, green, blue components and name them as matrices R, G and B. The ciphertext matrices R_2_, G_2_ and B_2_ can be obtained by using the algorithm in this paper. R_2_, G_2_ and B_2_ are separately the red, green and blue components of the final cipher image *C*.

## 5. Experimental Simulation and Security Analysis

In our experimental simulation, six gray images with size 256 × 256 are selected for test, namely, Cameraman, Lena, Rice, Pepper, all black and all white image. The key set of encryption system is the initial value of the chaotic system (2), which is set as {*x*_1_(0), *x*_2_(0), *x*_3_(0), *x*_4_(0), *x*_5_(0)} = {0.9654, 0.0546, 0.6705, 0.5698, 0.78546, 0.1854}. The algorithm is simulated in MATLAB 2016b.

### 5.1. The Encryption Effect

**Figure 5:**
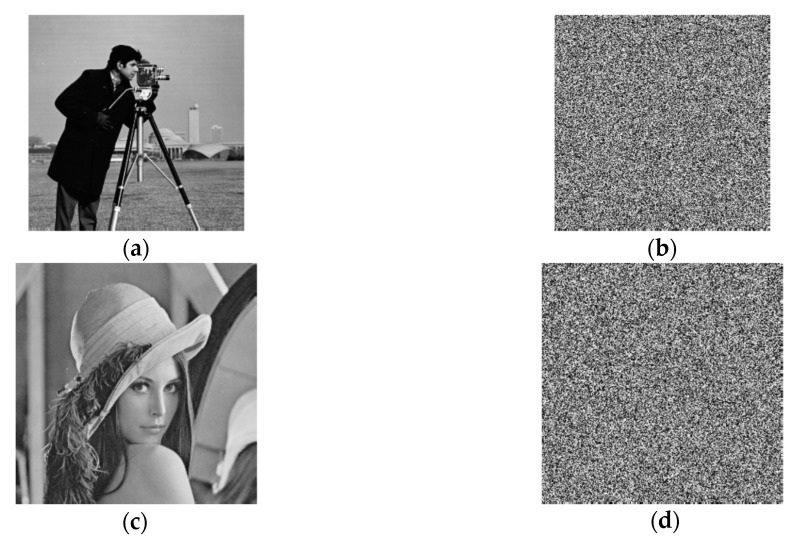

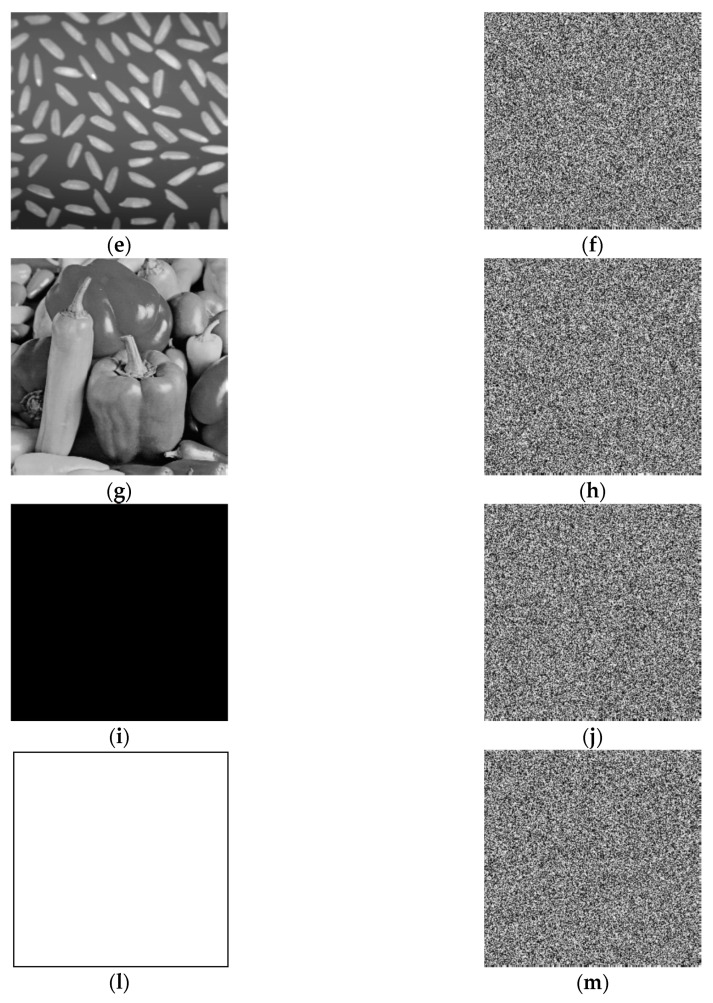
Simulation results. (**a**) Plaintext image Cameraman. (**b**) Encrypted image of (**a**). (**c**) Plaintext image Lena. (**d**) Encrypted image of (**c**). (**e**) Plaintext image Rice. (**f**) Encrypted image of (**e**). (**g**) Plaintext image Pepper. (**h**) Encrypted image of (**g**). (**i**) All black plaintext image. (**j**) Encrypted image of (**i**). (**l**) All white plaintext image. (**m**) Encrypted image of (**l**).

All the plain images and the encrypted images are shown in Figure 5. It can be seen that the encrypted images are in random noise style and no effective information can be obtained as it would be proven later on.

### 5.2. Key Space

A good image encryption algorithm should have enough key space to resist exhaustive attack. The key space of a cryptosystem should be at least 2^128^ to resist brute force attack. The key of the encryption algorithm proposed in this paper is the initial value of chaos system keys = {*x*_1_(0), *x*_2_(0), *x*_3_(0), *x*_4_(0), *x*_5_(0)}. The experimental results show that the accuracy of *x*_1_(0), *x*_2_(0), *x*_3_(0), *x*_4_(0), *x*_5_(0) can be 10^−15^. Therefore, the key space of the algorithm proposed in this paper is 10^75^ ≈ 2^24^^9^.

### 5.3. Statistical Characteristics Analysis

A secure encryption algorithm should be able to mask the statistical characteristics of plaintext. The statistical analysis of image includes histogram analysis and correlation coefficient analysis of adjacent pixels.

#### 5.3.1. Histogram Analysis

**Figure 6:**
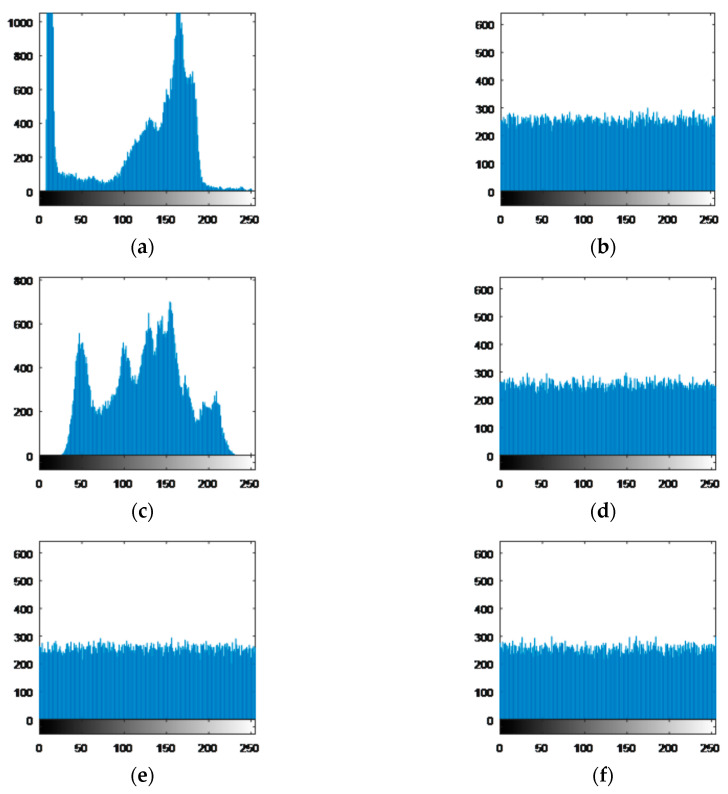
Histograms of plaintext image and corresponding ciphertext image. (**a**) The histogram of plaintext image “Cameraman.” (**b**) The histogram of ciphertext image of “Cameraman.” (**c**) The histogram of plaintext image “Lena.” (**d**) The histogram of ciphertext image of “Lena.” (**e**) The histogram of ciphertext image of all black. (**f**) The histogram of ciphertext image of all white.

The histogram of the image shows the distribution of the image pixel value. If the histogram distribution is more uniform and the statistical characteristic of the image is smaller, the corresponding encryption algorithm has stronger resistance to statistical attacks. Here, four images “Cameraman,” “Lena,” all black and all white are encrypted to verify the effectiveness of the algorithm in histogram. The histograms of plaintext and encrypted ciphertext images are shown in Figure 6a–f respectively. It can be seen that the histogram distributions of the encrypted images are almost uniform, which hide the statistical characteristics of the images.

The variance of histogram can also be used to measure whether it is evenly distributed. The smaller the variance value of image histogram, the more uniform the pixel value distribution of ciphertext image [37,38]. The variance of histogram can be calculated by

(28) $$\mathrm{var}(H)=\frac{1}{256^{2}}\sum_{j=1}^{256}\sum_{i=1}^{256}\frac{1}{2}{\left(h_{i}-h_{j}\right)}^{2}$$

where *H* = {*h*_1_, *h*_2_, …, *h*_256_}, *h_i_* and *h_j_* represent the number of pixels with gray values of *i* and *j*, respectively. By formula (28), the variance value of the plain histogram of the image “cameraman” is 110970, which proves that the number of plain image pixels in each gray level is out of balance, while the variance value of ciphertext histogram is about 280.203, which shows that the distribution of ciphertext images is uniform.

#### 5.3.2. Correlation Coefficient

In general, there is a strong correlation between adjacent pixels of plaintext image, while the correlation between adjacent pixels of ciphertext image is close to zero. In order to test the correlation between adjacent pixels, 2000 pairs of adjacent pixels are randomly selected from the original plaintext image and the encrypted image and the correlation coefficients between adjacent pixels are calculated in the horizontal direction, the vertical direction and the diagonal direction respectively as

(29) $$x_{c}=\frac{n\sum_{i=1}^{n}x_{i}y_{i}-\sum_{i=1}^{n}x_{i}\sum_{i=1}^{n}y_{i}}{\sqrt{n\sum_{i=1}^{n}x_{i}^{2}-{\left(\sum_{i=1}^{n}x_{i}\right)}^{2}}\sqrt{n\sum_{i=1}^{n}x_{i}^{2}-{\left(\sum_{i=1}^{n}x_{i}\right)}^{2}}}$$

Here, *x_i_* and *y_i_* are the pixel values of two adjacent pixel points, *n* represents the number of pixels. The calculation results of the six test images are shown in Table 3.

**Figure 7:**
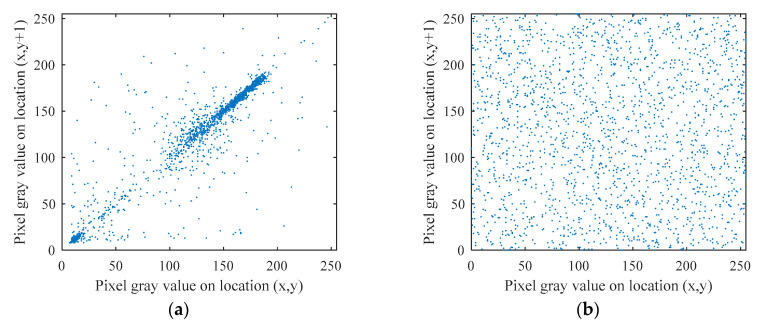

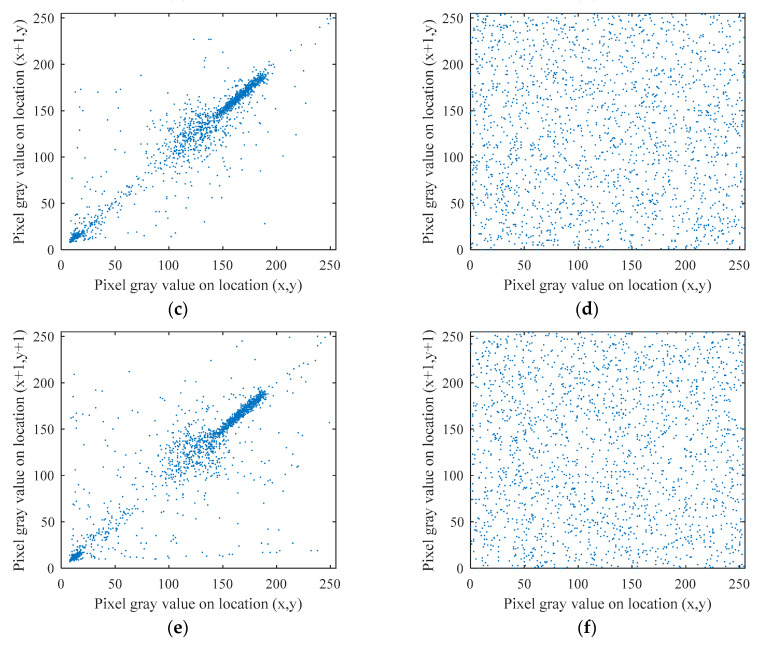
Distribution of adjacent pixels of the plain image Cameraman and its cipher image. (**a**) Horizontal direction of the plain image. (**b**) vertical direction of the plain image (**c**) diagonal direction of the plain image. (**d**) horizontal direction of the cipher image, (**e**) vertical direction of the cipher image. (**f**) diagonal direction of the cipher image.

Take “Cameraman” as an example, draw the correlation distribution map of the plain image and the corresponding cipher image in these three directions so as to compare the correlation between the adjacent pixels of the plain image. The results are shown in Figure 7.

From Table 3 and Figure 7, it can be seen that the adjacent pixels of plaintext image have strong linear relationship in three directions, while the adjacent pixels of ciphertext image show random relationship in three directions, which shows that the redundancy and correlation of pixels are removed.

#### 5.3.3. Information Entropy

Image information entropy is a quantitative reflection of the uncertainty of image information. The larger the entropy, the greater the uncertainty, that is, the stronger the randomness of image. Information entropy is represented by *E*

(30) $$E=-\sum_{i=0}^{255}p\left(i\right)\ln p\left(i\right)$$

where *p*(*i*) represents the probability of gray value *i*, that is, the proportion of the number of pixels with gray value *i* to all pixels in an image. The ideal value of entropy for an 8-bit gray-scale image is 8. The closer the value is to 8, the more uncertain the image is, the more uniform the distribution of image pixel value is. Table 4 shows the information entropy of cipher images of “Rice,” “Cameraman,” “Lena” and “Pepper” encrypted by this algorithm and other algorithms. Compared with other algorithms, this algorithm is closer to the ideal situation, that is, the encryption effect of this algorithm is better.

### 5.4. Sensitivity Analysis

#### 5.4.1. Key Sensitivity Analysis

**Figure 8:**
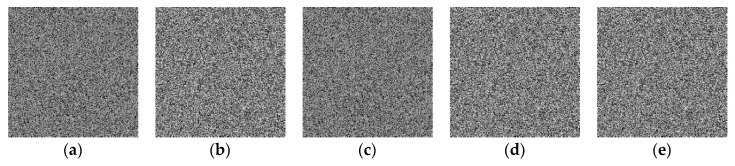
The image decrypted by the wrong key. (**a**) decryption result of key1. (**b**) decryption result of key2. (**c**) decryption result of key3. (**d**) decryption result of key4. (**e**) decryption result of key5.

A secure encryption algorithm should be sensitive to the key in order to resist brute force attacks. Key sensitivity means that if the decryption key is slightly different from the correct key, no useful information of the plaintext image can be obtained from the decryption result. We use the Key0 = (*x*(0), *y*(0), *z*(0), *u*(0), *w*(0)) = (10.656, 0.028, 0.059, 10.675, 0.023) to encrypt the original plaintext image “cameraman.” Then apply the following error keys Key1, Key2, Key3, Key4 and Key5 in the Table 5 to decrypt the ciphertext image and the decryption results are shown in Figure 8. It can be seen that no information of the original image can be obtained in the decrypted image, which also shows the high sensitivity of the algorithm to the key.

#### 5.4.2. Plaintext Sensitivity Analysis

If the same key is used to encrypt two plaint images with slight difference, the two cipher images obtained have great difference, which is called the algorithm is sensitive to plaintext. The algorithm’s sensitivity to plain image is the basis of resisting differential attack and chosen-plaintext attack. Number of Pixels Change Rate (NPCR) and Unified Average Changing Intensity (UACI) are commonly used to measure the sensitivity of encryption algorithms to plaintext. The formulas for the calculation of NPCR and UACI are as follows

(31) $$D(i,j)=\{\begin{array}{c} 1,if\;C_{1}\left(i,j\right)\neq C_{2}\left(i,j\right)\\ 0,f\;C_{1}\left(i,j\right)=C_{2}\left(i,j\right) \end{array}$$

(32) $$NPCR=\frac{1}{M\times N}\sum_{i=1}^{M}\sum_{j=1}^{N}D(i,j)\times 100\%$$

(33) $$UACI=\frac{1}{M\times N}\sum_{i=1}^{M}\sum_{j=1}^{N}\frac{\left|C_{1}\left(i,j\right)-C_{2}\left(i,j\right)\right|}{255}\times 100\%$$

where *M × N* is the size of the image. *C*_1_(*i*, *j*) represent the pixel in a coordinate (*i*, *j*) of the image and *C*_2_(*i*, *j*) represent the pixel in a coordinate (*i*, *j*) of another image. For 256 bit grayscale images, the expected values of *NPCR* and *UACI* are 99.6094% and 33.4635%, respectively [40].

To test the plaintext sensitivity, we use the following method: Firstly, we encrypt plain image P1 with a certain key K to get cipher image C1. Secondly, we randomly select a pixel from P1 and slightly change the value of the pixel, keep the value of other pixels unchanged and record the new image as P2. Then encrypt P2 with the same key K to get a new cipher image C2. we calculate the values of NPCR and UACI between C1 and C2. We have done 20 groups of test, in each test we randomly select one pixel in the plain image “Cameraman,” change its value with 1 bit and encrypt it. Finally, calculate the NPCR and UACI values between any two pairs of ciphertext image. The results are shown in Table 6. From Table 6, one can see that the NPCR and UACI values are very close to the ideal values.

### 5.5. Resistance to Typical Attacks

In the cryptanalysis, there are four typical attacks: (1) Ciphertext only attack: the attacker has no other auxiliary information except the intercepted ciphertext. Ciphertext only attack is the most common type of cryptanalysis and the most difficult one. (2) Known plaintext attack: the attacker owns part of plaintext and corresponding ciphertext at the same time and can break all or part of plaintext and key. (3) Chosen-plaintext attack: an attacker has a chance to temporarily gain access to the encryption machine, so he or she can choose some special plaintext and get the corresponding ciphertext, so as to decode all or part of the plaintext and key. (4) Chosen-ciphertext attack: the attacker temporarily obtains the use right of the decryptor, so he can decrypt any ciphertext and obtain the corresponding plaintext, so as to break the key.

Obviously, the chosen-plaintext attack is the strongest attack. If a cryptosystem can resist this attack, it must be able to resist the other three attacks. So only chosen-plaintext attack has been analyzed.

The security of the encryption scheme proposed in this paper mainly depends on the random sequence *CS*, *DS*, *K* and *R* and the generation of these random sequences is related to the sequence *S*. From Equations (4) and (17), we can see that the generation of *S* is related to every pixel of the plain image, so the random sequences *CS*, *DS*, *K* and *R* are related to the plaint image, that is, the secret key streams used to encrypt different images are different. On the other hand, the two rounds of diffusion mechanism further enhances the security of the algorithm, so it can resist the chosen plaintext (ciphertext) attack.

**Figure 9:**
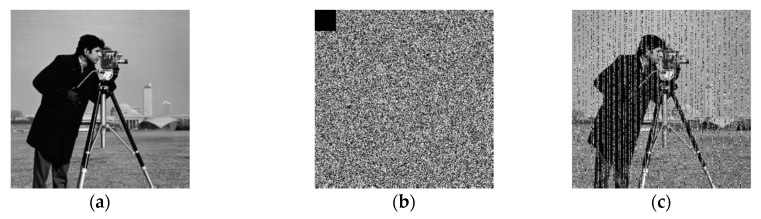
The decrypted image corresponding to the cut cipher image. (**a**) The plain image of cameraman. (**b**) The cipher image of cameraman with 32 × 32 pixels were cut. (**c**) The decrypted image of (**b**).

Because the algorithm is sensitive to plaintext, the disadvantage of the algorithm may be that the ability of ciphertext to resist noise, cut and compress attacks is weak. In Figure 9, we test the encrypted image of Cameraman with 32 × 32 pixels were cut, the decrypted image can be generally recognized. The error can propagate during the decryption process. The test results show that the decrypted image can be generally recognized if the error proportion of the pixels is about 1.5625%, which equal to (32 × 32)/(256 × 256).

### 5.6. Analysis of Algorithm Efficiency

The computation complexity is composed of measuring the number of operations and steps required to accomplish the encryption/decryption process and thus it is related with all the encryption processes. For a gray image that has *L* pixels, in the process of the confusion scheme, the sorting complexity is O (*L* × log*L*). The permutation operation is pixel level and its time complexity is *O* (*L*). For the DNA encoding and DNA decoding, the operation is bit level and each pixel has 8 bits, the time complexity is *O* (8*L*). Normally, *L* ≤ 1024 × 1024, log*L* ≤ 6.0206. Therefore, the total time complexity of the proposed scheme is *O* (8*L*). From the above analysis, it is evident that DNA encoding and DNA decoding of the plain image take more time.

## 6. Conclusions

Based on a new 5D continuous hyperchaotic system, an image encryption algorithm with DNA dynamic encoding mechanism is proposed in this paper. The algorithm consists of two stages: scrambling stage and two rounds of diffusion stage. The proposed algorithm not only has the advantages of “scrambling substitution” structure algorithm but also overcomes the difficulty of key management in “one time pad” encryption scheme and can resist chosen-plaintext attack.

The proposed image encryption algorithm has the following three advantages:

(1) In the diffusion stage, the dynamic rules of DNA encoding (decoding) are adopted, so the key streams used to encrypt different images are different and the algorithm can resist the attack of chosen plaintext (ciphertext).

(2) The algorithm has the effect of “one time pad” but the decryption key is only the initial value of the chaotic system, which overcomes the difficulty of key management in the “one time pad” encryption scheme (the key used to encrypt different plaintexts is different).

(3) Due to the two round diffusion mechanism, the algorithm is highly sensitive to plain image. Experimental results and theoretical analysis show that this algorithm can resist differential attack, brute-force attack, statistical attack and chosen-plaintext attack. Thus, the proposed algorithm has extraordinarily high security.

## Figures and Tables

**Table 1:** Eight rules of DNA encoding.

	1	2	3	4	5	6	7	8
00	A	A	C	G	C	G	T	T
01	C	G	A	A	T	T	C	G
10	G	C	T	T	A	A	G	C
11	T	T	G	C	G	C	A	A

**Table 2:** The DNA complementary property.

X	DNAcomplementnt(X)	X	DNAcomplementnt(X)
A	T	G	C
T	A	C	G

**Table 3:** Comparison of correlation coefficients between adjacent elements of images.

Images	Horizontal	Vertical	Diagonal
Plain image of “Cameraman”	0.9335	0.9592	0.9087
Cipher image of “Cameraman”	0.0070	−0.0004	0.0005
Plain image of “Lena”	0.9401	0.9695	0.9180
Cipher image of “Lena”	−0.0003	−0.0016	0.0022
Plain image of “Rice”	0.9776	0.9667	0.9585
Cipher image of “Rice”	−0.0149	−0.0022	0.0050
Plain image of “Pepper”	0.9429	0.9452	0.89721
Cipher image of “Pepper”	−0.0023	−0.0009	−0.0047
Plain image of “all black”	/	/	/
Cipher image of “all black”	0.0003	0.0046	−0.0044
Plain image of “all white”	/	/	/
Cipher image of “all white”	0.0002	−0.0051	−0.0021

**Table 4:** Information entropy of ciphertext image.

Images	Ours	Ref. [39]	Ref. [24]	Ref. [35]
Rice	7.9972	7.9965	7.9945	7.9970
Cameraman	7.9974	7.9987	7.9921	7.9976
Lena	7.9973	7.9973	7.9968	7.9972
Pepper	7.9974	7.9929	7.9934	7.9987

**Table 5:** Error keys used in decryption.

Keys	*x*(0)	*y*(0)	*z*(0)	*u*(0)	*w*(0)
Keys1	10.656 + 10^−15^	0.028	0.059	10.675	0.023
Keys2	10.656	0.028 + 10^−15^	0.059	10.675	0.023
Keys3	10.656	0.028	0.059 + 10^−15^	10.675	0.023
Keys4	10.656	0.028	0.059	10.675 + 10^−15^	0.023
Keys5	10.656	0.028	0.059	10.675	0.023 + 10^−15^

**Table 6:** Comparison of plaintext sensitivity between our algorithm and other algorithms.

Algorithms	*NPCR* (%)		*UACI* (%)	
	Min	Average	Min	Average
Ours	96.70	99.61	33.48	33.67
Ref. [24]	94.70	99.58	33.28	33.37
Ref. [35]	99.57	99.72	33.52	33.64
Ref. [39]	99.53	99.67	33.32	33.49
